# Are studies of human gut microbiome the new fad following the SNP mainstream?

**DOI:** 10.20945/2359-3997000000568

**Published:** 2022-11-28

**Authors:** Sandra Roberta Ferreira Vivolo, Gabriel da Rocha Fernandes

**Affiliations:** 1 Universidade de São Paulo Faculdade de Saúde Pública Departamento de Epidemiologia São Paulo SP Brasil Departamento de Epidemiologia, Faculdade de Saúde Pública, Universidade de São Paulo, São Paulo, SP, Brasil; 2 Fiocruz Minas Instituto Rene Rachou Grupo de Informática de Biossistemas Belo Horizonte MG Brasil Grupo de Informática de Biossistemas, Instituto Rene Rachou, Fiocruz Minas, Belo Horizonte, MG, Brasil

**DEAR EDITOR,**

During the “pre-Human Genome Project (HGP) era” that precedes the 1990s, the availability of genetic sequencing technologies has triggered a boom of studies in literature that searched for genetic variations associated with a variety of normal and pathological conditions in humans. Hundreds of thousands of reports dealing with single nucleotide polymorphisms in the human DNA were identified adding, valuable knowledge for health sciences (1). This scenario forced the scientific community to become familiar with the approaches to assess the genomic information and infer associations to phenotypes. Many candidate genes identified are now helpful for disease prediction and for intervention purposes. In parallel, there was a growing interest in unveiling the entire human genome and the expectation that this would bring light to the genesis of diseases and therapeutic strategies. The HGP and Celera Genomics data assembled sequences comprising 3.4 billion nitrogenous bases from the human DNA (2,3), but the main goal of discovering mechanisms of diseases to improve quality of life was incompletely achieved.

In XXI century, the increased availability of genetic sequencing equipment was a powerful tool for looking at the microbiome inhabiting the human body and allowed great achievement of knowledge by mapping the microbial DNA. Like the HGP (2,3), a research effort occurred to determine its composition involving trillions of microorganisms, and a consortium organized by the National Institutes of Health developed the Human Microbiome Project (HMP) and mapped the microbiome of healthy humans (4).

Using the MeSH “microbiome” of animal models or humans in PubMed, results are impressive. Are studies of human microbiomes the new fad following the SNP mainstream addressed to deepen our knowledge on health and diseases? They should be considering the need of understanding the physiological dialogue “host-microbiota” since our birth.

In analogy to that experienced with the genes and polymorphisms reports, which required specific knowledge by non-experts about approaches and terms from genetics, genomics, and bioinformatics, now it is time to add concepts from microbiology and ecology to understand the microbiome universe better. This 12-year-old HMP has been widely used to designate specific groups of microorganisms as the cause of multifactorial diseases. From lab-derived animals and mechanistic experiments, a long way of investigations must be followed to understand such a complex ecosystem. Comparing the stability of the genome to the microbiome, the latter is highly impacted by environment, lifestyle, diet, and health status, being it challenging to point it as a cause or a consequence of diseases.

This letter has absolutely no intention to fill gaps in colleagues' knowledge but perhaps motivate a minimal comprehension of practical terms in readings involving the gut microbiota:

- Different aims and limitations among study approaches, such as metagenomics and metataxonomics;
- Ecological concepts such as richness, alpha and beta-diversity, and their interpretations;
- Identification of differentially abundant organisms, prevalence, and reproducibility of this kind of result;
- Usage of taxonomic or functional biomarkers for diagnostics and prognostics prediction.

Before reaching the clinical practice, translational research and well-designed experiments should be conducted to clarify the role of the gut microbiota in a disease etiopathogenesis and its relative importance among other clinical and habits variables. The best evidence reported in microbiota studies will come from multidisciplinary research teams that aggregate diverse and complementary knowledge, recognizing the impossibility to handle all the aspects, from methodological and pathophysiological to clinical issues.
